# Revitalizing the common peroneal function index for assessing functional recovery following nerve injury

**DOI:** 10.1002/brb3.1968

**Published:** 2020-12-13

**Authors:** Calder Fontaine, Eric A. Yeager, Michael Sledziona, Amanda K. Jones, Jonathan Cheetham

**Affiliations:** ^1^ Department of Clinical Sciences Cornell College of Veterinary Medicine Cornell University Ithaca NY USA

**Keywords:** common peroneal nerve, index, regeneration

## Abstract

**Background and Aims:**

Peripheral nerve injury is common with poor functional recovery and consequent high personal and societal costs. Sciatic nerve transection and assessment of recovery using sciatic functional index (SFI) are widely used. SFI is biologically limited as axonal misdirection of axons supplying flexors and extensors in the hindlimb, after nerve injury can lead to synkinetic innervation and function which does not correspond to the degree of axonal regeneration.

**Methods:**

We reevaluated the use of traditional metrics such as print length (PL), toe spread (TS), and intermediate toe spread (ITS) as well as hock angle at mid‐swing as approaches for determining recovery. We used two alternative approaches in discrete cohorts of rats following common peroneal crush injury, transection with repair and critical gap, using transection with ligation as a negative control. We compared walking track analysis (print) with digital capture and kinematics.

**Results:**

PL, TS, and ITS varied as expected after injury. The traditional functional index for common peroneal injury using inked prints failed to describe recovery and we derived new indices to describe recovery (all *R* ^2^ > 0.88, *p* < .0001) although pre‐injury PFI was never attained by any of the models. Kinematic analysis identified hock angle at mid‐swing as a useful predictor of recovery (*p* < .0001).

**Interpretation:**

Using complementary approaches.

## Graphical abstract

Sciatic nerve transection and assessment of recovery using sciatic functional index (SFI) is widely used. SFI is biologically limited as axonal misdirection of axons supplying flexors and extensors in the hindlimb, after nerve injury can lead to synkinetic innervation and function which does not correspond to the degree of axonal regeneration. We identify metrics that can be used to determine the time to recovery and the degree of recovery after common peroneal crush and transection without the biological limitations associated with SFI.

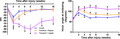

## 1 INTRODUCTION

Peripheral nerve injuries (PNI) are common, with an annual incidence of 360,000 in the United States (Dayawansa et al., 2014), high personal and societal costs and a high requirement for support services and/or rehabilitation (Noble et al., 1998; Selecki et al., 1982). Repair after PNI is further worsened by age (Tanaka et al., 1992; Vaughan, 1992; Verdú et al., 1995) and a delay prior to repair (Sulaiman and Gordon, 2009; Gordon et al., 2011; Furey et al., 2007).

The most commonly used paradigm to determine the effects of experimental novel treatments is the sciatic nerve of the experimental rat and the corresponding longitudinal metric the sciatic functional index (SFI) (Wood et al., 2011). SFI arose from the initial observation that walking tracks made by rats have consistent and reliably quantified characteristics (Hruska et al., 1979).

De Medinaceli, et al. later developed specific measurements for print length (PL, distance from heel to digit III), total toe spread (TS, the distance between digits I and V), intermediate toe spread (IT, the distance between digits II and IV), and distance to the opposite foot (TOF, distance from digit III of the measured print to digit III of the next contralateral print) (Medinaceli et al., 1982). The longest measurements were taken of the prints from both the experimental (surgical) limb (E) and the contralateral (C) limb, and the contralateral limb measurements were used to normalize experimental limb measurements into “factors” using a variant of percent change that de Medinaceli called “percent deficit.” These factors were weighted equally to produce a formula that resulted in Sciatic Function Index (SFI) which scaled nerve function between 0 and −100; 0 representing perfect nerve function and −100 reflected complete nerve dysfunction.

Sciatic nerve transection models have consistently shown a relatively small degree of observable functional recovery when estimated using SFI, limiting their usefulness in peripheral nerve studies (Wood et al., 2011). This is partially due to the mixed grouping of fascicles that contribute to the tibial and common peroneal branches of the sciatic nerve, supplying flexors and extensors combined with axonal misdirection during the regenerative process. Axonal misdirection leads to non‐selective reinnervation of the distal stump fascicles causing a mixture of tibial and common peroneal branches entering the distal stump and altered activation patterns of opposing muscle groups during locomotion (Gramsbergen et al., 2000).

As a refinement to SFI, Bain‐Mackinnon‐Hunter (BMH) (Bain et al., 1989) later revised the de Medinaceli SFI formula, and introduced similarly derived formulae for the Tibial Function Index (TFI) and Peroneal Function Index (PFI) for use with the sciatic nerve's distal branches. Using PFI mitigates the limitation imposed on SFI as a result of axonal misdirection, since the motor units of the common peroneal nerve have related (rather than opposing) muscular functions.

The use of PFI (Ruiter et al., 2007; Chen et al., 2009; Wong et al., 2011; Hare et al., 1992) is less common than SFI (Bittner et al., 2012; Chen et al., 2007, 2009, 2010; Clavijo‐Alvarez et al., 2007; Dinh et al., 2009; Hare et al., 1992; Kim et al., 2007; Lee et al., 2013; Matsuda et al., 2010; Meek et al., 2007; Monte‐Raso et al., 2006, 2008; Nagao et al., 2011; Nie et al., 2007; Penna et al., 2012; Ruiter et al., 2007; Whitlock et al., 2009; Wong et al., 2011; Wood et al., 2010; Xu et al., 2012), and several problems with walking tracks are still apparent in the literature, such as contracture, related to loss of function of tarsal flexor/digital extensor muscles (Chen et al., 2009; Hare et al., 1992; Wong et al., 2011), or other causes of poor print quality (Maeda et al., 1993; Medinaceli et al., 1982; Meek et al., 1996). Other problems have been suggested, but not investigated, such as alteration of contralateral limb prints as a result of compensatory strategies during locomotion (Hare et al., 1992), and the effect of velocity on walking track analysis (Shenaq et al., 1989; Varejão et al., 2001; Walker et al., 1994). Finally, one previously unaddressed problem with traditional walking track analysis is its use of maximal, rather than average, lengths for each measured component (Bain et al., 1989; Medinaceli et al., 1984).

Here, we used a range of acute common peroneal nerve injury models to observe changes in the components traditionally used for walking track analysis (PL, TS, and ITS) and also evaluated ankle angle during mid‐swing and mid stance, following injury. We used these data to propose revised formulae for calculating recovery after peripheral nerve injury and produce an approach that allows assessment of time to recovery as well as degree of recovery after varying degrees of PNI.

## 2 MATERIALS AND METHODS

### 2.1 Ethics statement

This study was performed in accordance with the Public Health Service Policy on Humane Care and Use of Laboratory Animals (PHS Policy on Humane Care & Use of Laboratory Animals, 2002), the NIH guide for Care and Use of Laboratory Animals (Guide for the Care & Use of Laboratory Animals, 2011), and federal and state regulations. It was approved by the Cornell University Institutional Animal Care and Use Committee (IACUC, protocol #2012‐0099). ARRIVE guidelines for reporting in vivo experiments were used throughout (Kilkenny et al., 2010).

### 2.2 Animal care

Sprague Dawley or Lewis rats (The Jackson Laboratory, Bar Harbor, ME), aged 12 weeks, were used in this study. Animals were given a 72‐hr acclimatization period prior to any procedure. All animals were maintained in a temperature and light‐controlled environment (12‐hr light/dark cycle) and were permitted ad libitum access to water and standard laboratory rodent food (Tekland Mouse Breeder Diet; Harlan Laboratories, Madison, WI).

### 2.3 Experimental design

Two distinct studies were conducted. The first (transection and repair and critical gap) evaluated a traditional approach using inking of the hind feet then allowing rats to traverse a substrate upon which they leave their hind paw prints (Hruska et al., 1979; Lowdon et al., 1988; Medinaceli et al., 1982; Shen & Zhu, 1995; Zellem et al., 1989). In a second study kinematic analysis was used to evaluate gait after crush, transection and repair and transection with ligation. Assessment of locomotive function in both studies was carried out using a corridor apparatus composed of transparent Plexiglas(R) with an enclosed black Plexiglas(R) box at the end that allowed rats to climb down into their own cage. Video was recorded, using a Canon EOS Rebel T4i (Canon USA, Inc.), at a resolution of 1,920 × 1,088, a frame rate of 60 fps, and a high shutter speed (1/640s, f4.5, ISO800) to minimize blurring between frames. Kinematic analysis and gait tracking were performed using SIMI^®^ gait tracking software (Simi Reality Motion Systems GmbH, Unterschleissheim, Germany). Rats were trained for 3 weeks prior to their baseline (pre‐injury) assessment as pilot experiments had demonstrated an improvement in performance with training. A minimum of three assessments were obtained for each rat at each time point. Rejection of a trial occurred for if the rat paused or stopped in the middle of the trial or the prints made were unusable due to poor inking or other process‐related error.

### 2.4 Surgical procedures

All surgical procedures were performed under general anesthesia (1.5%–2.0% isoflurane in oxygen) using a stereo microscope (M60 modular stereo microscope; Leica Microsystems, Buffalo Grove, IL) adapted for surgical use. All animals received meloxicam (0.5 mg/kg; Nordbrook, Inc.), administered subcutaneously, perioperatively and at 24 hr post‐operatively. Post‐operatively, a bittering agent (Bitter Apple^®^, Grannick's Bitter Apple Company, Norwalk, CT) was applied twice daily to deter autotomy.

#### 2.4.1 Transection

The common peroneal (CP) nerve branch was identified and transected 3 mm distal to its division from the sciatic nerve with immediate coaptation using two interrupted epineurial sutures of 9–0 Ethilon™ (Ethicon, Inc., Somerville, NJ). Care was taken to maintain the rotational relationship of the severed stumps.

#### 2.4.2 Critical Gap

A critical gap nerve injury was created by suturing the proximal and distal stumps of the CP into a 17 mm silicone conduit, with a gap of 15 mm between the stumps (Lundborg et al., 1982).

#### 2.4.3 Crush

Crush was performed using a titanium aneurism clip (Sugita, Mizuho Corporation) to provide a crush force of 1.35N over a 1.0 mm section of nerve for 30 s.

#### 2.4.4 Transect and Ligate

Proximal and distal CP stumps were ligated with 9/0 ethilon after transection.

### 2.5 Group allocation

Groups were allocated as follows: Study 1, paper‐substrate assessment, Sprague Dawley rats, transection and repair (*n* = 12), critical gap (*n* = 6) assessments at baseline and 1, 2, 3, 4, 8, 12, 16, 20, and 24 weeks post‐injury; Study 2, kinematic assessment, Lewis rats, transection and repair (*n* = 8), crush (*n* = 8), transect and ligate (*n* = 7), assessments at baseline and 2, 4, 8, 12, and 16 weeks post‐injury.

### 2.6 Functional assessment: Paper

Walking track assessments were obtained by inking the rat's feet, using an oversized inking pad (Ranger Industries), and then permitting them to run across a single 43 cm long by 10 cm wide piece of plain white paper was placed on the floor of the corridor apparatus.

### 2.7 Functional assessment: Kinematic

Prior to baseline assessment, animals were anesthetized, the left hind limb clipped, and hair removed using a depilatory cream (Nare™). The following points were tattooed using a commercial tattoo gun with black ink: Stifle, tip of calcaneus, head of the fourth metatarsal, pads of digits 2 and 4.

### 2.8 Measurement, calculation, and statistical analysis

**Figure 1:**
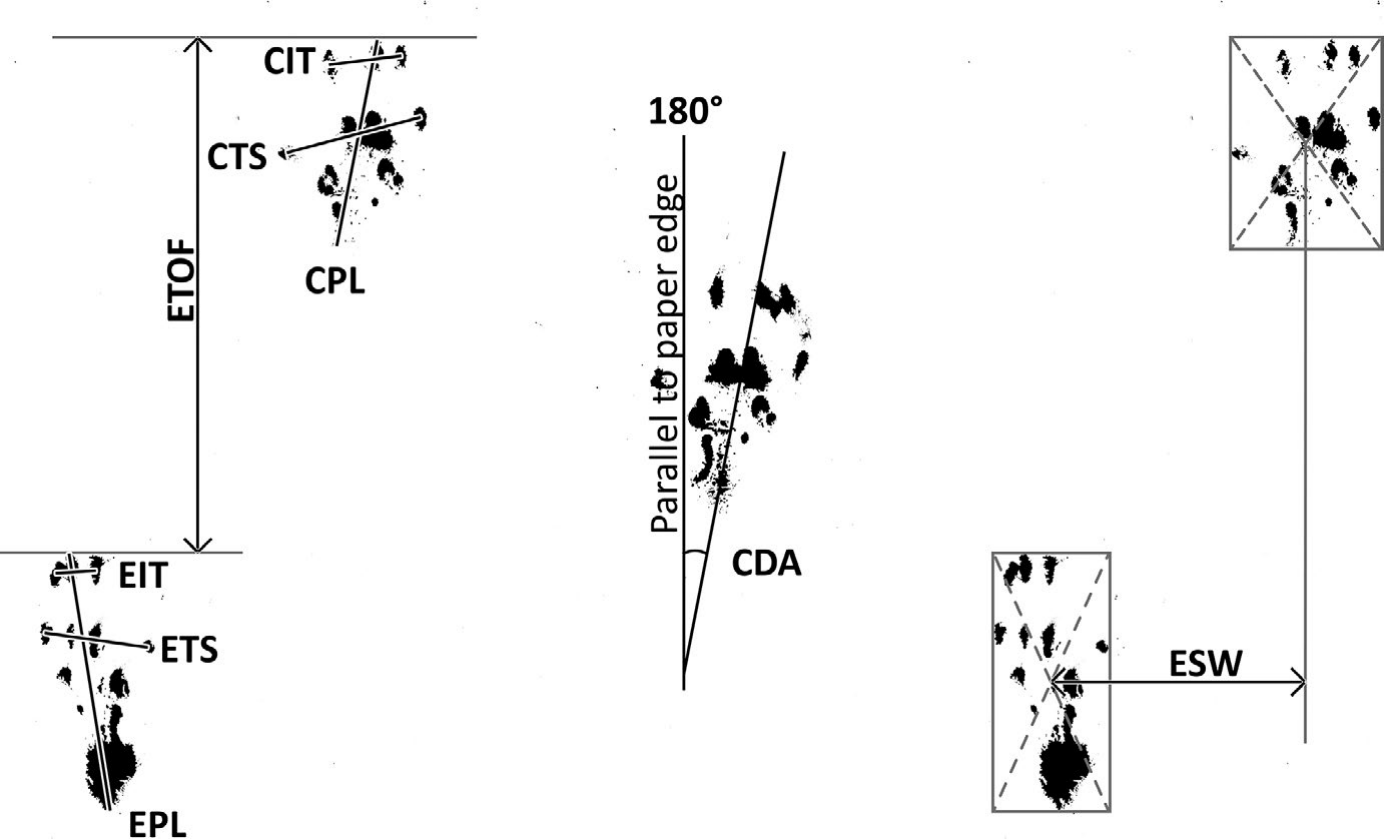
Component Measurements for Walking Track Analysis. The methods for measuring each component of walking track analysis for both the left, experimental (E), and right, contralateral (C), limbs: print length (PL), toe spread (TS), intermediate toe spread (IT), distance to the opposite foot (TOF), deviation angle (DA), and stance width (SW) are shown. Note that CTOF is not shown here but would be measured in a similar manner to ETOF, except from the right print to the next sequential left print. Similarly, DA is shown only for the contralateral limb (CDA) and SW is shown only for the experimental limb (ESW)

Walking tracks were scanned into digital images (CanoScan LiDE 110, Canon USA, Inc), and measurements were made of each component for both the experimental (surgical) and contralateral limbs (Figure 1), using Photoshop CS6^®^ software (Adobe Systems, Inc.). The measurements taken included print length (PL, the distance between cranial extent of digit III and caudal extent of the print), the total spread (TS, the distance between the centers of paw pad I and paw pad V), intermediate toe spread (IT, the distance between the centers of paw pad II and paw pad IV), and distance to the opposite foot (TOF, the vertical distance between the cranial aspects of contralateral prints). Measurements not previously described included deviation angle (DA, the angle of the foot with respect to the edge of the paper; the top of the page = 180°) and stance width (SW, the horizontal distance between the print and the next contralateral print). All measurements for all prints were recorded for each trial (usually 2 prints per limb). Two sets of measurements were then composed: (a) a “trial maximum” set, derived from the largest measurement for each component and (b) a “trial average” set, derived from the average measurement of each component from all ipsilateral prints.

Traditional PFI was calculated using the trial maximum data and the BMH formula (Bain et al., 1989). Baseline (pre‐injury) values were averaged for each walking track components of the trial maximum data set, and a variant of the BMH PFI was calculated using baseline (pre‐injury) normalization for PL and TS and the BMH equation. Baseline (pre‐injury) averages were also calculated for the trial averaged data set and both the traditional BMH PFI and the baseline (pre‐injury) variant BMH PFI methods of calculation were then applied to these data. Finally, velocity was calculated using the recorded time data and the known distances between the photoreceptor sensors on the corridor apparatus.

The effect of time on each measured component, the calculated indices and calculated velocities for each trial was evaluated using ANOVA and Tukey's HSD. Multiple linear regression analysis was used to determine the effect of time on baseline (pre‐injury) normalized PFI factors calculated for each of the trial averaged components, with time treated as a categorical variable to allow for non‐linear effects of time.

Baseline (pre‐injury) data were determined to represent “normal” function. Week 3 data were determined to represent maximum dysfunction for the PL, TS, IT, and TOF factors, and week 4 data were determined to represent maximum dysfunction for the DA and SW factors. Therefore, week 0 was designated as “0” and weeks 3 and 4 were both assigned as ‘−100’ to create the independent axis for regression analysis. Each of the factors was then screened for effect on the index, using only data from weeks 0, 3, and 4.

Subsequently, three independent models were constructed using multiple linear regression based on the levels of significance of the effect of individual components (*p* < .05, *p* < .004 and *p* < .0001) and using the complete data set (all time points). Finally, the new PFI was calculated for each of the three models for each trial at all time points.

All statistical analyses were carried out using JMP software (SAS Institute, Cary, NC). The data that support the findings of this study are available from the corresponding author upon reasonable request.

## 3 RESULTS

### 3.1 Paper trial

Exclusions: One rat was euthanized prematurely, due to autotomy of its injured limb.

*Speed*: There was a significant decrease in velocity of rats moving across the corridor apparatus over time (*p* < .0001, ANOVA, Figure S1).

#### 3.1.1 Functional index components

There were significant differences between time points for each of the measured components (PL, TS, IT, TOF, DA, and SW) for experimental limb, regardless of whether the maximum value or the average value for each trial was compared (*p* < .0001, all components). For the contralateral limb, IT (*p* = .042) and SW (*p* < .0001) had statistically distinct groups for the trial maximum data set, whereas PL (*p* = .021) and SW (*p* < .0001) had significantly different groups for the trial average data set (data not shown).

#### 3.1.2 Calculated factors

Baseline (pre‐injury) normalization of each component for the experimental limb revealed significant differences over time in PL, TS, IT, TOF, DA, and SW using both the trial maxima and trial average data sets (all *p* < .0001, ANOVA, Figure S2 and S3). For the contralateral limb, there were significant differences between groups only for the trial average PL and SW factors (*p* = .02 and *p* < .0001, respectively, ANOVA, Figure S3) and for the trial maximum IT and SW factors (*p* = .038 and *p* < .0001, respectively, ANOVA, Figure S3).

Some of the trial averaged data set factors calculated for the experimental limb, such as PL, TOF, and DA, differed from their trial maximum counterparts (Figure S2). However, some of the experimental limb factors, such as TS, IT, and SW, differed little between the trial averaged and trial maximum data sets (Figure S2). For the contralateral limb, all factors were very similar, whether derived from the trial maximum or trial averaged data set (Figure S3). The exception was SW, in which trial averaged derived values deviated ~3%–5% more than the trial maximum data (Figure S3).

#### 3.1.3 Calculated peroneal function index

Peroneal function index (PFI) calculated using the BMH equation varied significantly over time. This was true when using contralateral limb normalized factors derived from either trial maximum measurements (BMH, *p* < .0001, ANOVA, Figure S4A), or trial average measurements (BMH, *p* < .0001, ANOVA, Figure S4B). Similarly, there were significant differences between time points when we used baseline (pre‐injury) normalized factors derived from either trial maximum measurements (Baseline, *p* < .0001, ANOVA, Figure S4A), or trial average measurements (Baseline, *p* < .0001, ANOVA, Figure S4B). As expected, the standard error of the mean (*SEM*) was smaller for PFI derived from the trial average data set. Contrary to expectation, however, the PFI increased (for all methods of calculation), contrary to previous reports, immediately post‐surgery and gradually decreased over the experimental time frame. PFI for the trial averaged data set was observably higher for all post‐surgical time points, using either contralateral limb or baseline (pre‐injury) normalization.

#### 3.1.4 New peroneal function index for paper‐based assessment

We then developed a new model for peroneal function index (PFI) for use with paper assessment. Three independent models were constructed based on significance levels obtained by screening CP PFI factors. Screening revealed that only a subset of the factors was correlated with changes observed between week 0 and weeks 3 and 4 following transection. ETS, EDA, EPL, and CDA significantly contributed (all *p* < .0001 all, Table 1), as did CPL, ETOF, and ESW (*p* = .001, *p* = .004, and *p* = .035, respectively, Table 1). Velocity did not significantly contribute to any model (*p* = .058, Table 1).

**Table 1:** Significant components for revised PFI formula

Component		*p*‐value
Experimental limb Toe Spread (ETS)	<.0001	
Experimental limb Deviation Angle (EDA)	<.0001	
Experimental limb Print Length (EPL)	<.0001	
Contralateral limb Deviation Angle (CDA)	<.0001	
Contralateral limb Print Length (CPL)	.001	
Experimental limb distance to Top of Opposite Foot (ETOF)	.004	
Experimental limb Stance Width (ESW)	.035	
Velocity	.058	
Contralateral limb distance to Top of Opposite Foot (CTOF)	.081	
Experimental limb Intermediate Toe spread (EIT)	.21	
Contralateral limb total Toe Spread (CTS)	.60	
Contralateral limb Intermediate Toe spread (CIT)	.75	
Contralateral limb Stance Width (CSW)	.92	

Note

*p*‐values for individual components resulting from model screening. Three models were constructed based on significance levels: (1) all with significant *p*‐values, (2) those with *p*‐values < .004, and 3) those with *p*‐values < .0001. Velocity was determined not to contribute significantly to any model (*p* = .058).

The first model (7 factors, Model 1, Figure S5) included all components with a significant *p*‐value (EPL, ETS, EDA, ETOF, CPL, CDA, and ESW). The second model (5 factors, Model 2) included only those components with a p‐value less than 0.004 (EPL, ETS, EDA, CPL and CDA). The third model (4 factors, Model 3) included only those components with significant p‐values less than 0.0001 (EPL, ETS, EDA, and CDA). All models had p‐values < 0.0001 and high adjusted *R* ^2^ values (Model 1:0.89, Model 2:0.89, and Model 3:0.88). PFI was then calculated for all time points using each of the three new models.

**Figure 2:**
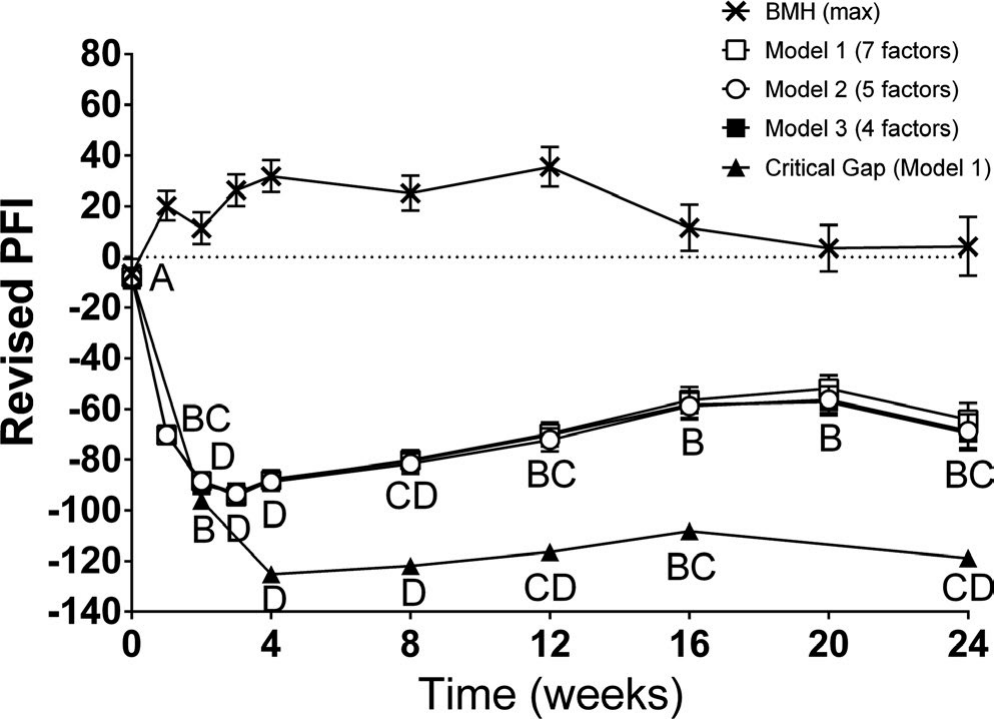
Comparisons of new calculated models of PFI. There was a significant effect of time for all three new models of PFI for acute transection and coaptation (Model 1, 2, and 3; *p* < .0001 for all). All models had similar patterns of near zero pre‐injury PFI, nadir PFI at week 3, increasing PFI trends through week 20 and a drop in PFI at week 24. Similarly, the critical gap PFI showed a significant decrease through week 4 followed by increasing function through week 20. Letters represent distinct groups based on Tukey's HSD test (groups not connected by the same letter are significantly distinct) for Model 1 only (other models were similar). Data shown are the mean and standard error (*SEM*). Shown with BMH PFI for comparison (no letters assigned)

PFI varied significantly over time using each of the new models (*p* < .0001 for all three models, ANOVA, Figure 2). For each of the models, PFI decreased immediately following surgery to a nadir at week 3, followed by a gradual increase in PFI from week 3 through week 20, although pre‐injury PFI was never attained by any of the models.

#### 3.1.5 Critical nerve gap

Walking track analysis following surgical transection with a critical common peroneal nerve gap, using Model 1 (7 factors), validated the new PFI model revealed a loss of function reaching a nadir at week 4 followed by a small, but significant recovery over the remainder of the experimental time frame (Figure 2).

### 3.2 Video trial

*Exclusions:* No animals were excluded.

*Speed:* There was no effect of time after injury on speed across the apparatus in any group (all *p* > .2).

#### 3.2.1 Functional index components

**Figure 3:**
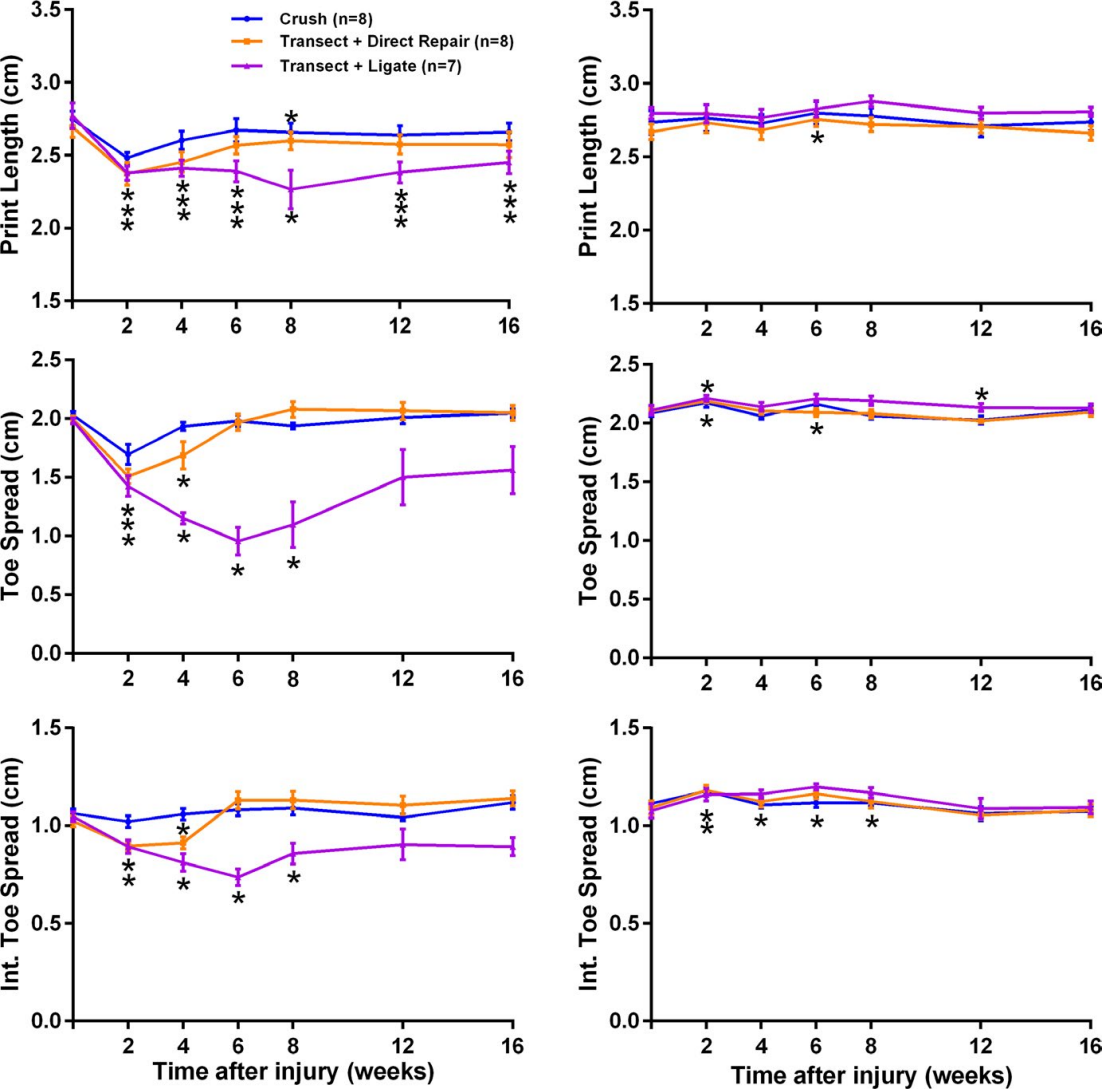
Print length, toe spread, and intermediate toe spread detect common peroneal nerve injury and track recovery. Similar and significant decreases in PL, TS, and ITS were observed following all three injury types (all *p* < .001). These parameters were able to detect the more rapid recovery in the crush model compared to the transection model. Data shown are the mean and standard error (*SEM*), * indicates significantly different to pre‐injury data, Dunnet's test, (*p* < .05)

TS in the experimental limb significantly decreased after injury in all three groups (ligation, *p* = .004; transect and coaptation, *p* < .0001; and crush *p* = .0001, Figure 3) before recovering in repaired (6 weeks) and crushed (4 weeks) animals but not the ligated group. ITS decreased after injury after ligation (*p* = .006), transection, and coaptation (*p* < .0001) but not following crush injury (*p* = .2); before recovering in repaired (6 weeks) and crushed (4 weeks) animals but not the ligated group. PL significantly decreased, in contrast to the paper study, following ligation (*p* = .006) but not after transection with repair (*p* = .07) or crush (*p* = .12).

In the control limb, injury produced a small but significant increase in TS after transection and repair (*p* = .02) and crush (*p* = .02), but not ligation (*p* = .29, Figure 3). ITS also increased slightly in the control limb after crush (*p* = .03), ligation (*p* = .03) but not repair (*p* < .14). Contralateral PL only increased in the crush group (*p* = .02).

#### 3.2.2 Calculated peroneal function index

**Figure 4:**
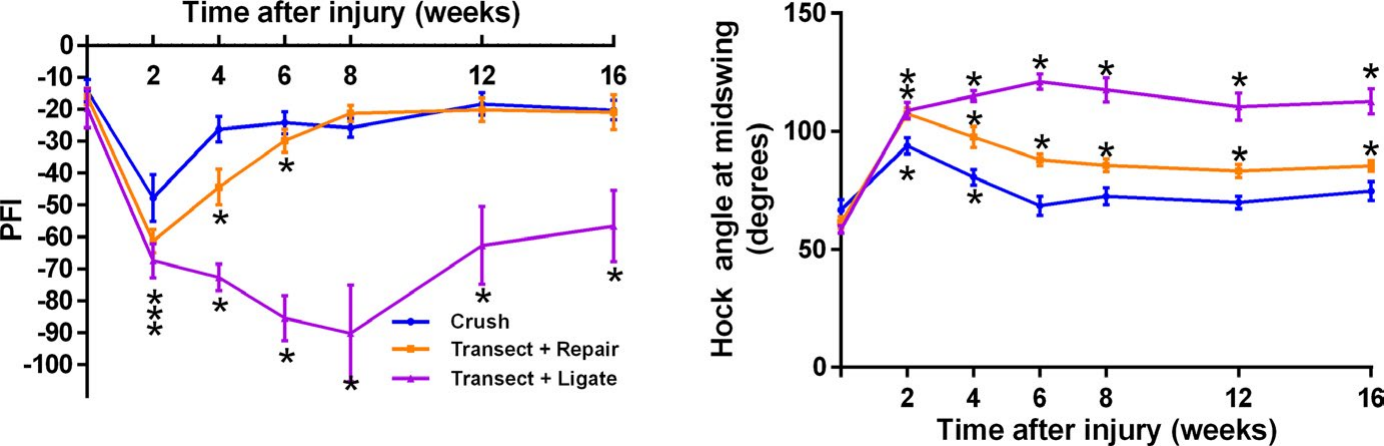
Traditional calculation of PFI and hock angle at mid‐swing track recovery after CP injury. PFI demonstrates more rapid recovery after crush than transection and repair. Similar and significant decreases in PFI and hock angle at mid‐swing were observed following all three injury types (all *p* < .001) with recovery by 4 and 8 weeks using PFI and 6 weeks after crush injury using hock angle at mid‐swing. Hock angle at mid‐swing did not return to baseline values in either of the transected groups. Data shown are the mean and standard error (*SEM*), * indicates significantly different to pre‐injury data, Dunnet's test, (*p* < .05)

Peroneal function index (PFI) was significantly reduced in all three injury types (all *p* < .001, Figure 4) when calculated using the BMH equation which only includes TS and PL. As anticipated, PFI recovered in the crush group by 4 weeks, the repaired group by 8 weeks and did not recover in the ligated group.

#### 3.2.3 Hock angle

Hock angle at mid‐swing increased significantly after injury, as anticipated following disabling of the *tibialis anterior* muscle which flexes the hock during the swing phase (figure 4, all *p* < .0001). Mid‐swing hock angle showed a faster and high degree of recovery after crush injury than after transection. No recovery was observed in the ligated group.

## 4 DISCUSSION

Peroneal function index (PFI) is far less common in the literature than its sciatic predecessor (SFI) (Wood et al., 2011). Crush injury models that use PFI to evaluate functional recovery in rats generally demonstrate loss of function immediately following injury followed by recovery over time that approaches, but does not achieve, pre‐injury levels of function (Ruiter et al., (2007); Wong et al., 2011; Hare et al., 1992). However, common peroneal transection models have been sparsely used with inconsistent results, sometimes showing expected trends of loss followed by recovery of function (Chen et al., (2009)) while in other studies producing variable results (Hare et al., 1992).

Here, we present two alternative approaches to determine return to function after common peroneal nerve crush or transection and repair. The kinematic approach combining print parameters and gait data (hock angle at mid‐swing) provides a robust, straightforward assessment of the return to function after common peroneal nerve crush or transection. Toe spread returned to pre‐injury values by 6 weeks in the crush group and 8 weeks in the transection and repair group, but not in the negative control of the transected and ligated group. Similarly hock angle plateaued at 80+% of pre‐injury values by 6 weeks in the crush group and 8 weeks in the transection and repair group, but not in the negative control (transect and ligate). We include the paper‐based substrate study data as it extends previous work in the field and kinematic analysis is not available to all workers in this area.

It is important to note that the original BMH formula does not capture or describe injury and recovery after injury correctly, as the print length is artifactually increased due to the heel strike occurs much earlier than the toes strike so artifactually elongating the print. This artifact is avoided with kinematic capture. As a result, in the initial work with a paper‐based substrate, high‐quality prints were obtained but applying the previously reported BMH PFI to these measurements produced an interpretation that was not consistent with the basic underlying biological processes of injury and repair, despite individually measured components demonstrating clear differences over time that were consistent with loss and recovery of function.

Two problems were identified in the previously described methods used for walking track analysis: (Bain et al., 1989; Medinaceli et al., 1982) first, using the extreme (in this case, the longest) of each measurement for each trial, and second, using contralateral limb measurements to normalize surgical limb measurements. Since the common peroneal nerve is responsible for motor innervation of muscles that provide tarsal flexion and digital extension, longer measurements for toe spread (TS) and intermediate toe spread (IT) would reflect increases in function, whereas longer print length (PL) measurements would reflect loss of function (Dyce et al., (2010)). Secondly, it is very likely that compensatory limb loading would result in altered prints from the uninjured contralateral limb. Indeed, by comparing print measurements from all time points for the contralateral limb to pre‐injury prints, we show that there are significant differences in the contralateral limb's prints post‐injury. Therefore, these prints are unreliable as a standard by which to normalize the surgical limb prints over time.

First, we evaluated the effect of using average measurements instead of maxima for each trial and using pre‐injury (baseline) measurements instead of contralateral limb measurements to normalize surgical limb measurements. However, the BMH PFI model still produced results that were not biologically sensible, suggesting an increase in function post‐injury followed by a time‐dependent loss of function toward baseline. Therefore, we proposed the formulation of a revised functional index for common peroneal transection injury models.

In order to develop the most rigorous revised model, we evaluated print measures that had been excluded previously when modeling the original PFI (TOF, DA, and SW). We used average measurements for each trial and used pre‐injury measurements to normalize both surgical and contralateral limb measurements. We also included velocity in our screening process and demonstrated that velocity was not significant in any model. All three models described (five, six, and seven factors) suggest a loss of function at the time of injury and a gradual recovery of function over time, which is consistent with expectations. Furthermore, the final model (using seven factors) provided consistent results when applied to a critical gap transection model of the common peroneal nerve (Figure 2 and Prest et al., 2018). However, using the scale described by this model, loss of function was greater for critical gap transections than for direct‐repaired transections, with week 4 nadir PFIs for critical gap repaired transections measuring approximately −125, compared to a nadir PFI of −95 for direct‐repaired transections. The degree of recovery over the experimental time frame was small, but significant.

In summary, we proposed two new approaches to determine recovery after common peroneal nerve injury in the rat. Using a paper‐based substrate, we present three models which use trial average data, pre‐injury normalized measurements for both the surgical and contralateral limbs and consider affected measurements from both of these limbs. In two separate models of rats with transected common peroneal nerves, these models reveal a loss of function at the time of injury and gradual recovery of function over time, which is consistent with expectations. In the kinematic dataset, we present metrics using hock angle and PFI components which track recovery after crush and transection with low variability. These revised approaches for determining common peroneal function are expected to provide a more consistent and reliable estimation of functional recovery in common peroneal transection models in rats and allow quantification of regenerative approaches to PNI, allowing assessment of time to recovery as well as overall recovery.

## CONFLICT OF INTEREST

The authors declare no competing financial interests.

## AUTHOR CONTRIBUTIONS

Calder Fontaine, Eric Yeager, Michael Sledziona, and Amanda Jones performed the study. Eric Yeager, Calder Fontaine, and Jonathan Cheetham conceived and designed the study. All authors analyzed the data and wrote the paper.

### Peer Review

The peer review history for this article is available at https://publons.com/publon/10.1002/brb3.1968.

## Supporting information

Fig S1

Fig S2

Fig S3

Fig S4

Fig S5

## Data Availability

The data that support the findings of this study are available from the corresponding author upon reasonable request.
