# Supplementary figures and images for: Revitalizing the common peroneal function index for assessing functional recovery following nerve injury
Source: Brain Behav. 2020 Dec 13;11(2):e01968. doi: 10.1002/brb3.1968 (PMC7882187; doi:10.1002/brb3.1968)

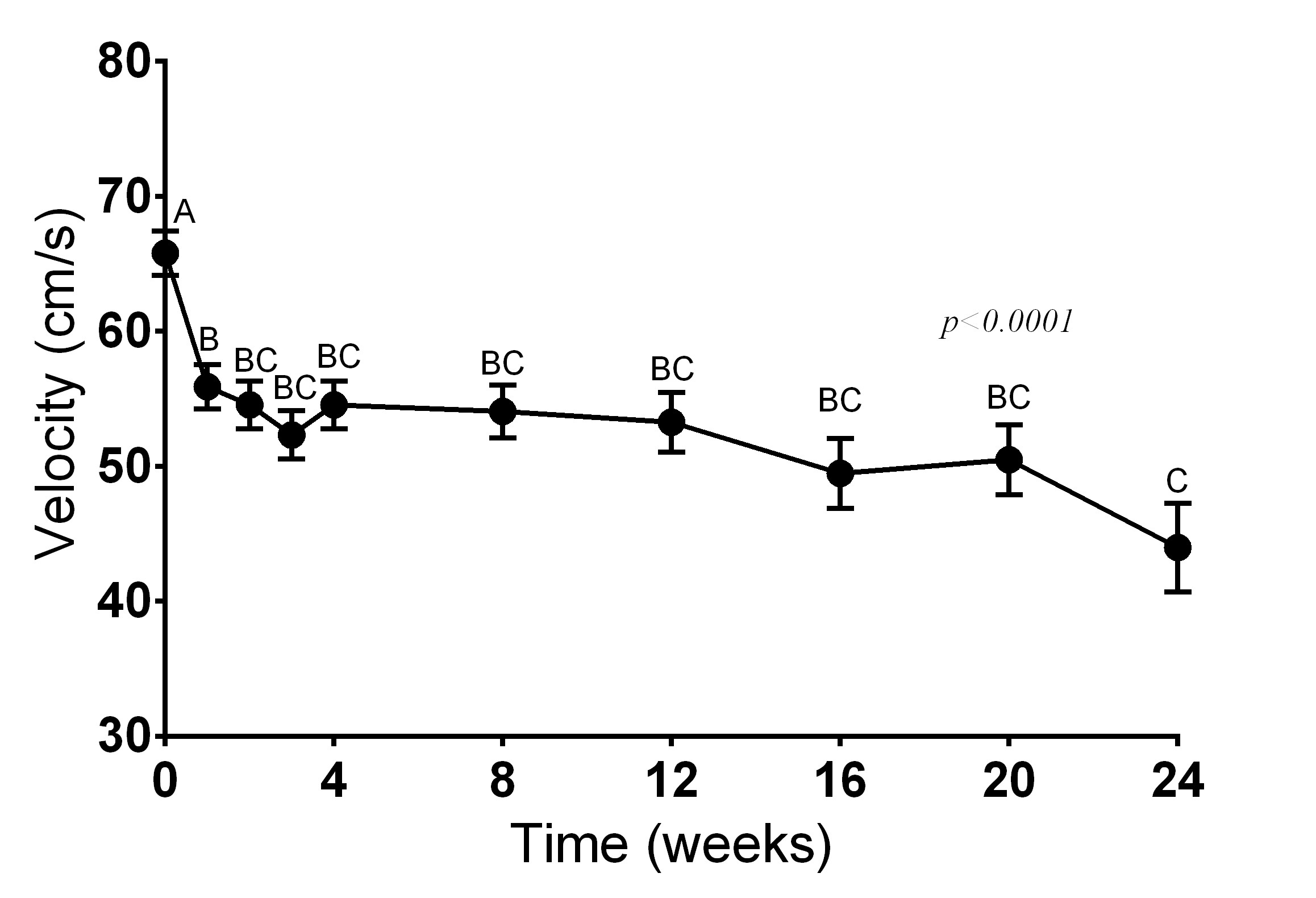

Supplement: Supplementary file 1 — Fig S1 [file BRB3-11-e01968-s001.jpg]

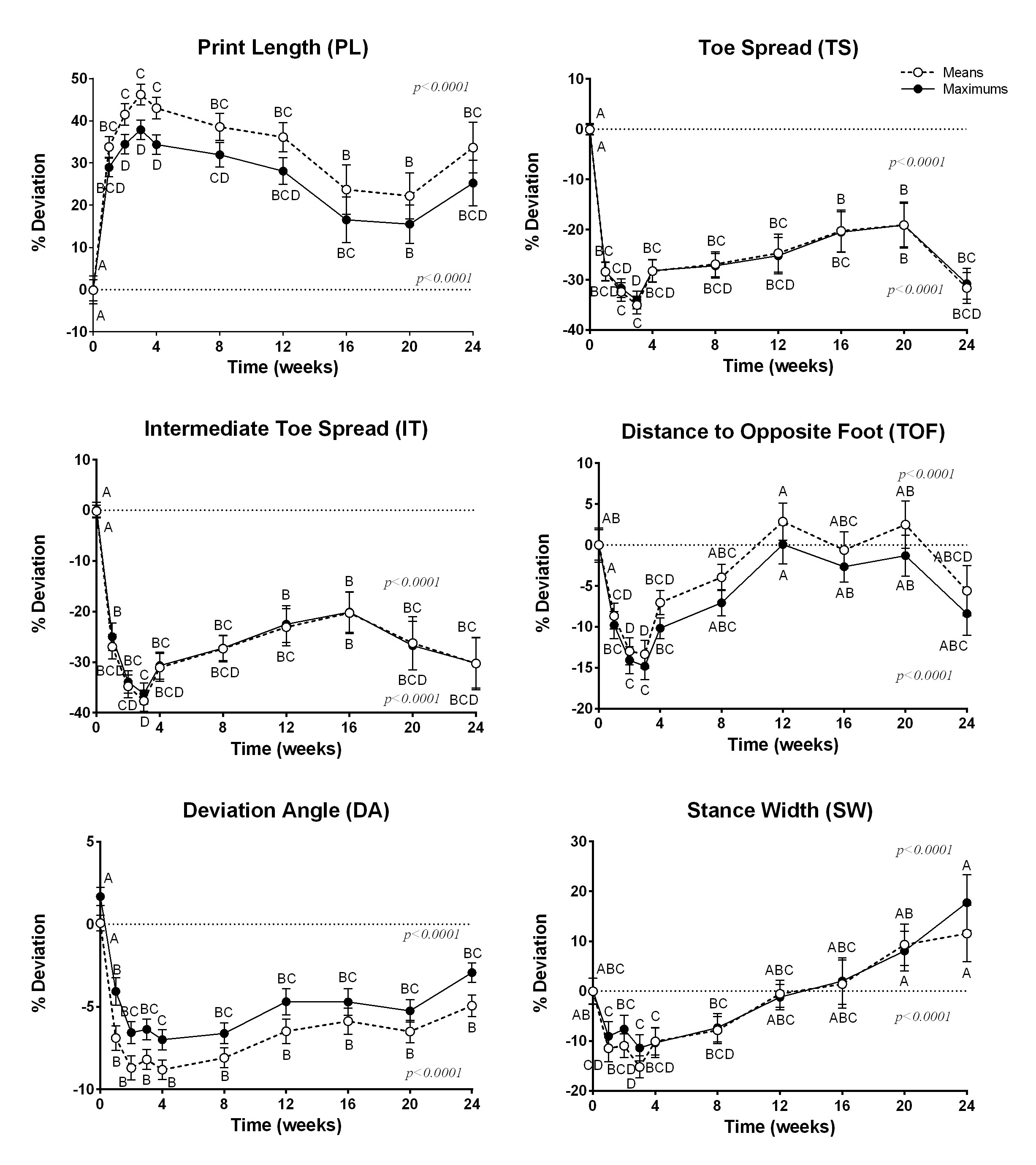

Supplement: Supplementary file 2 — Fig S2 [file BRB3-11-e01968-s002.jpg]

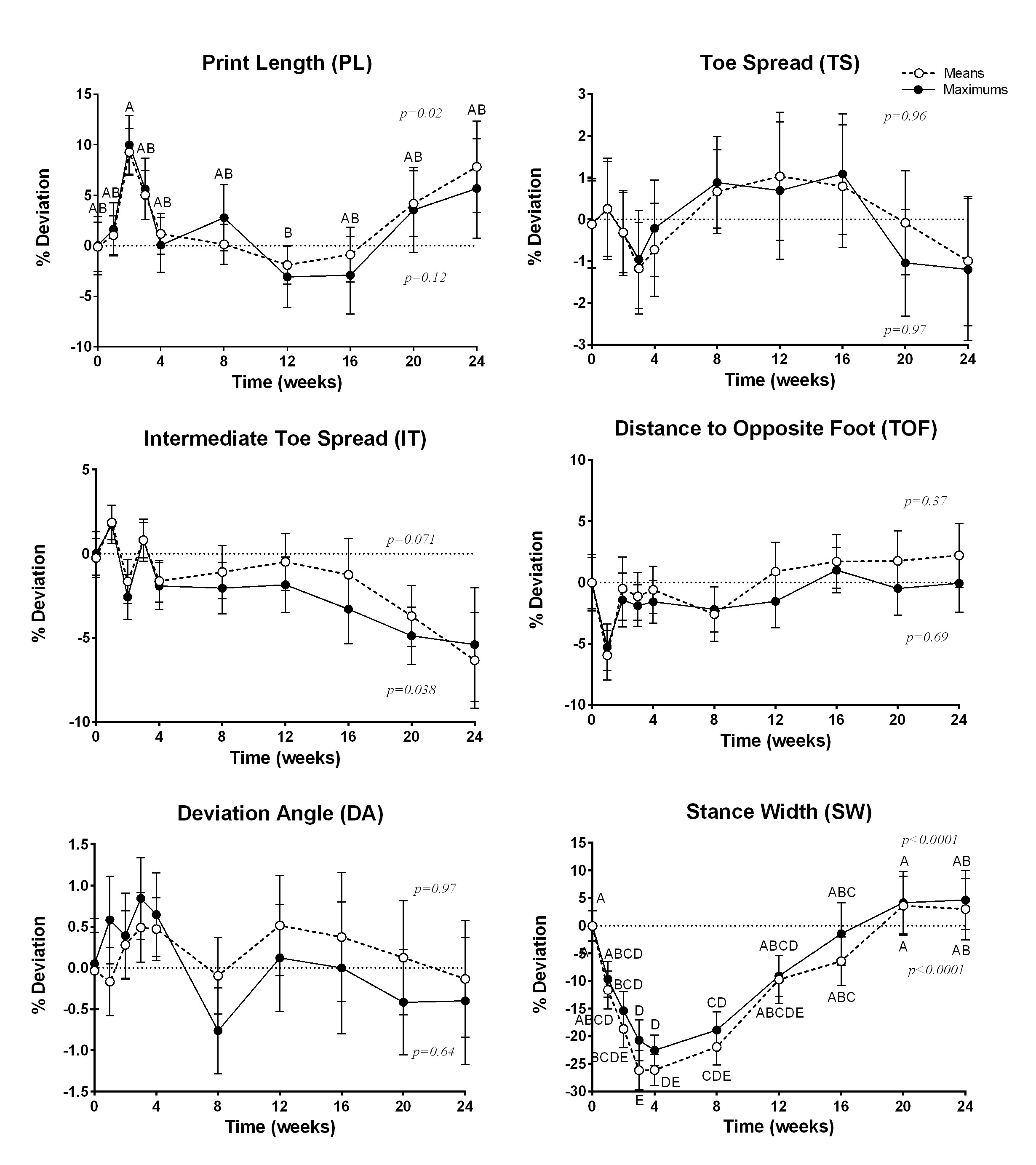

Supplement: Supplementary file 3 — Fig S3 [file BRB3-11-e01968-s003.jpg]

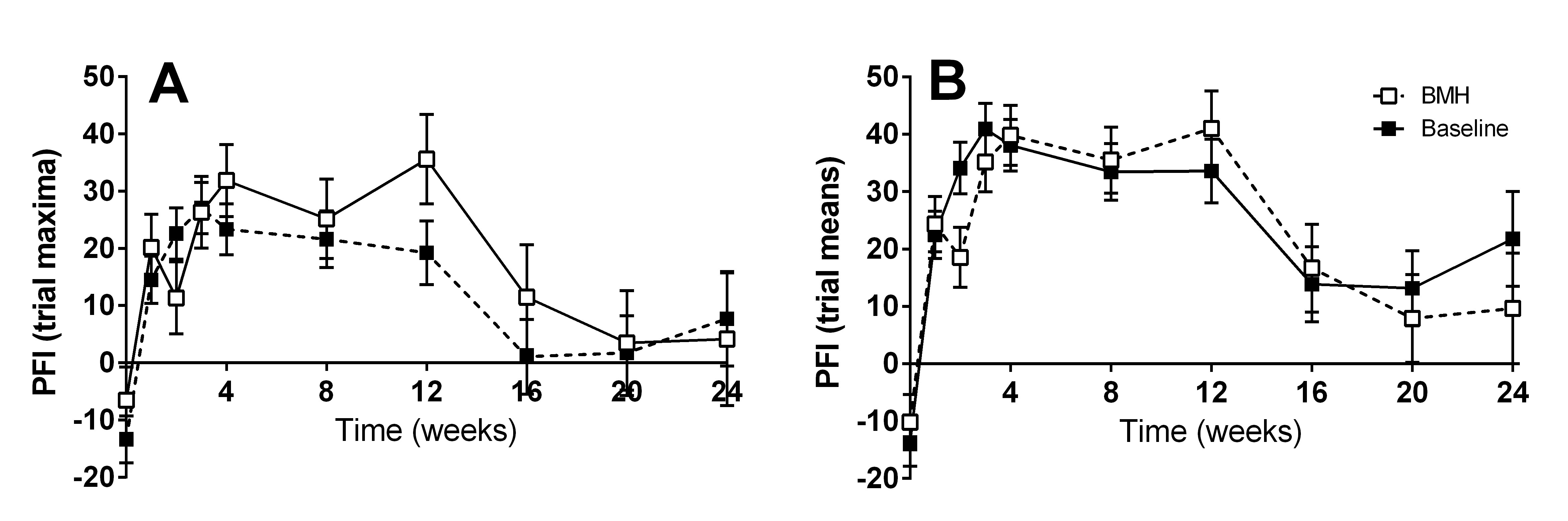

Supplement: Supplementary file 4 — Fig S4 [file BRB3-11-e01968-s004.jpg]

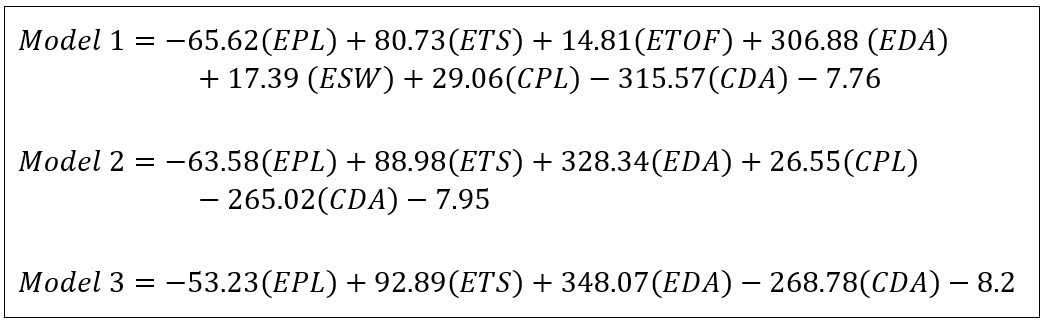

Supplement: Supplementary file 5 — Fig S5 [file BRB3-11-e01968-s005.jpg]
